# International Collaboration in Primary Ciliary Dyskinesia

**DOI:** 10.1002/ppul.71888

**Published:** 2026-10-28

**Authors:** Petra Pennekamp, Michele Manion, Heymut Omran

**Affiliations:** ^1^ Department of General Pediatrics University Children's Hospital Muenster Muenster Germany; ^2^ European Reference Network for Rare Respiratory Diseases (ERN‐LUNG) Frankfurt am Main Germany; ^3^ PCD Foundation Minneapolis Minnesota USA

**Keywords:** international network, Primary Ciliary Dyskinesia, rare disease, registry

## Abstract

Primary Ciliary Dyskinesia (PCD) exemplifies how international collaboration can transform the study of a rare disease. Since the identification of the first PCD‐associated genes in 1999–2002, parallel developments in Europe and North America, driven by patient organizations, academic investigators, and coordinated funding initiatives, have progressively converged into a unified global research infrastructure. Early milestones included the founding of patient advocacy groups, the NIH‐funded Genetic Disorders of Mucociliary Clearance Consortium, and European projects such as EUCILIA and SYSCILIA, which respectively established coordinated clinical research infrastructure and advanced ciliary systems biology. The 2012 EU‐funded BESTCILIA project formalized transatlantic collaboration, establishing the International PCD Registry and iPCD Cohort, generating the first robust diagnostic evidence base, and enabling the first multinational randomized controlled trial in PCD. This infrastructure was subsequently sustained and expanded through the BEAT‐PCD COST Action and its successor ERS Clinical Research Collaboration, the ERN‐LUNG PCD Core Network, and the PCD Clinical Trial Network, alongside parallel North American developments including the PCD Foundation Clinical and Research Centers and PCDF Network for Clinical Trials. Together, these initiatives have harmonized diagnostic pathways, produced joint ERS/ATS diagnostic guidelines, and created durable platforms for multinational trials and registries. Dedicated scientific meetings, culminating in the planned 2026 Global PCD Congress, further embody this convergence. Despite this progress, disparities in global representation and diagnostic access remain, underscoring the need for continued international coordination as the field moves toward precision medicine approaches.

## 1 Rare Diseases and International Collaboration

Rare diseases pose a unique challenge to healthcare and biomedical research. While each condition is individually uncommon, together they affect hundreds of millions of people globally and constitute a substantial, yet often under‐recognized, burden on patients, families, and healthcare systems [1]. Their low prevalence, coupled with marked clinical and genetic heterogeneity, has traditionally limited opportunities for systematic investigation and the generation of high‐quality evidence. Consequently, advances in diagnosis, disease characterization, and therapeutic development increasingly rely on collaborative research that extends beyond institutional and national boundaries [2, 3]. By facilitating the integration of clinical expertise, genomic resources, and longitudinal patient data, international networks have transformed the study of many rare disorders and have become indispensable for conducting adequately powered research and clinical trials [4]. These principles are especially relevant for ciliopathies, a group of genetically diverse multisystem disorders in which global collaboration has been instrumental in expanding knowledge of disease biology, improving diagnostic yield, and establishing the foundations for future therapeutic strategies [5, 6].

### 1.1 Search Methodology

This narrative review draws on the authors' first‐hand knowledge of and involvement in the field, complemented by targeted literature searches to identify and verify relevant sources. Details of the search strategy are provided in Supporting Information S1.

### 1.2 History of Primary Ciliary Dyskinesia and Kartagener's Syndrome

Primary Ciliary Dyskinesia (PCD, ORPHA:244) is a rare, genetically and clinically heterogeneous group of disorders categorized as motile ciliopathies [7, 8, 9]. Its clinical recognition began in the early 20th century with the description of bronchiectasis, chronic sinusitis, and *situs inversus totalis*, later termed Kartagener's syndrome, by AK Siewert in 1903 [10] and Manes Kartagener in 1933 [11]. The underlying cause remained debated until the 1970s, when electron microscopy by Björn Afzelius revealed absent dynein arms in the motile cilia of affected patients [12, 13, 14, 15, 16, 17, 18, 19, 20], establishing the link between ciliary ultrastructural defects, bronchiectasis, and left‐right organ asymmetry. The turn of the millennium brought a paradigm shift in PCD research.

The first PCD‐associated gene, *DNAI1*, was identified by Pennarun et al. in 1999 in a 9‐year‐old boy with classic PCD, situs solitus, and absent outer dynein arms [21]. Around the same time, Omran et al. [22] mapped a new PCD locus to chromosome 5p15–p14 in a consanguineous family with absent outer dynein arms and Kartagener syndrome. Within this region, they identified *DNAH5* as a strong candidate gene and subsequently found *DNAH5* mutations in eight families, including seven individuals with Kartagener syndrome [23]. The presence of affected individuals with and without *situs inversus* within the same families demonstrated randomization of left–right asymmetry, establishing *DNAH5* as the first gene associated with PCD and Kartagener syndrome [23]. These studies, together with animal studies of the left‐right organizer, revealed the mechanical role of motile monocilia in breaking embryonic symmetry via Nodal signaling [24, 25, 26, 27, 28, 29, 30, 31].

The subsequent integration of high‐speed video microscopy [32], immunofluorescence screening [33, 34, 35, 36] and high‐throughput genetic analysis [37, 38] has led to the identification of currently over 60 genes associated with PCD [39]. These advancements have transformed the understanding of *situs inversus* from an anatomical curiosity into a developmental disorder rooted in ciliary dysfunction, illustrating a successful translational trajectory from bedside clinical observation to molecular genetic characterization [7, 8].

### 1.3 International Collaborations That Shaped Modern PCD Research

The transformation of PCD from a rare disease managed by a few expert centers into an internationally coordinated research field was driven by the parallel development of collaborative networks in North America and Europe. Though these initiatives arose independently, shaped by different healthcare systems and funding mechanisms, they built on longstanding transatlantic collaborations among individual investigators and addressed similar challenges: improving diagnosis, understanding disease mechanisms, building longitudinal cohorts, developing evidence‐based care standards, and creating trial infrastructure. Over time these efforts became increasingly interconnected, forming today's global PCD research community (Figure 1).

**FIGURE 1:**
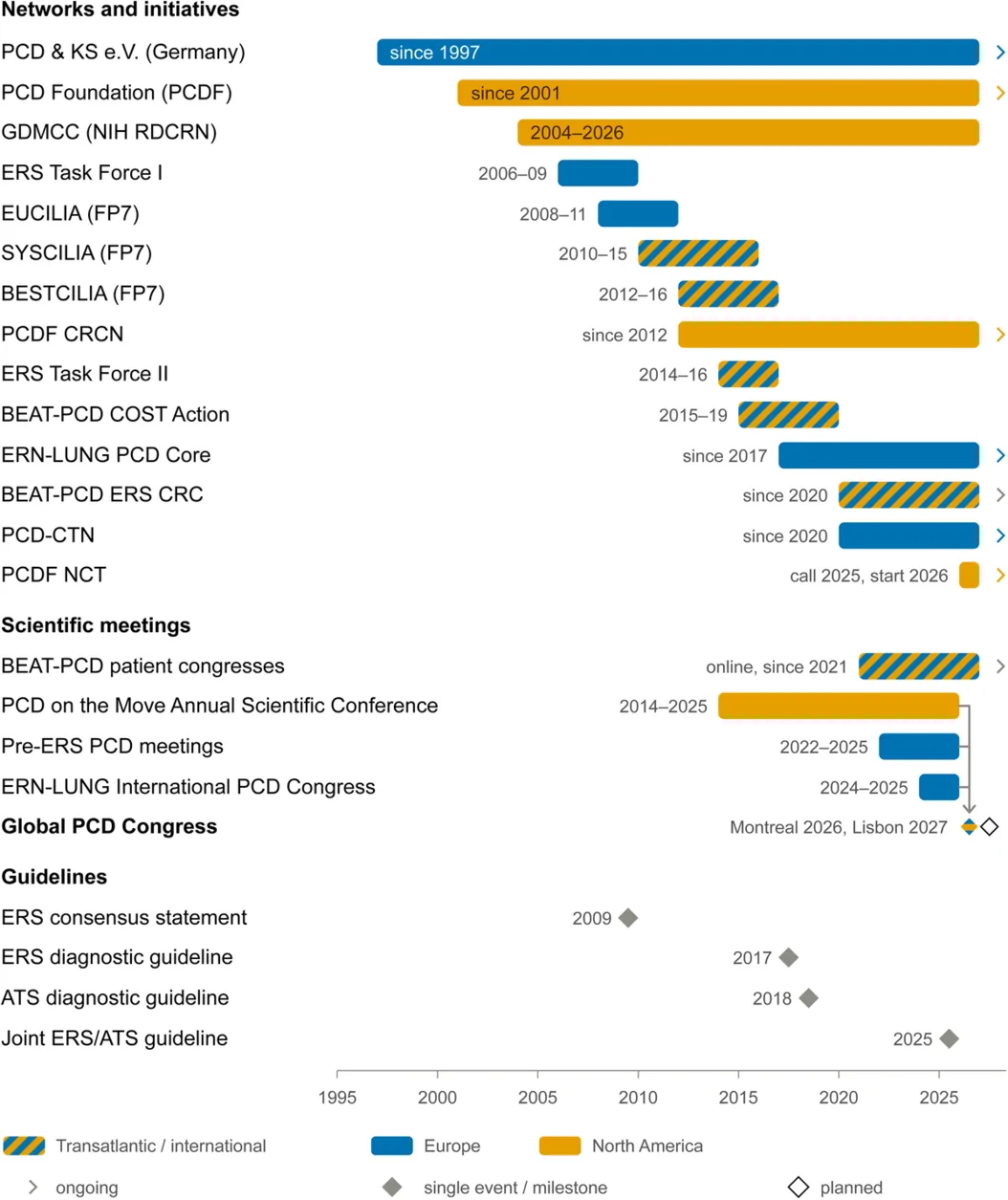
Timeline of international collaboration in Primary Ciliary Dyskinesia (PCD). Bars indicate the duration of networks, projects and meeting series; arrows indicate ongoing activities; filled diamonds mark single events and guideline publications; the open diamond marks the planned second Global PCD Congress (Lisbon, 2027). Colors indicate geographical scope: European (blue), North American (orange), and transatlantic/international (blue‐orange stripes). The PCDF CRCN was established in 2012 to provide patients with access to specialized PCD diagnosis and care. After accreditation procedures, training and a vetting process had been set up, the first site was accredited in 2015. Pre‐ERS PCD meetings were jointly organized by the ERS Clinical Research Collaboration BEAT‐PCD and the ERN‐LUNG PCD Core. The ERN‐LUNG International PCD Congress bar shows in‐person congresses only (Cyprus 2024, Naples 2025); preceding online meetings are not shown. From 2026, PCD on the Move, the pre‐ERS PCD meetings and the ERN‐LUNG International PCD Congress were merged into the Global PCD Congress (connecting lines), jointly organized by the PCD Foundation, the ERN‐LUNG PCD Core and the ERS CRC BEAT‐PCD. ATS, American Thoracic Society; COST, European Cooperation in Science and Technology; CRC, Clinical Research Collaboration; CRCN, Clinical and Research Centers Network; CTN, Clinical Trial Network; ERN, European Reference Network; ERS, European Respiratory Society; FP7, EU Seventh Framework Programme; GDMCC, Genetic Disorders of Mucociliary Clearance Consortium; KS, Kartagener syndrome; NCT, Network for Clinical Trials; NIH, National Institutes of Health; PCDF, PCD Foundation; RDCRN, Rare Diseases Clinical Research Network. [Color figure can be viewed at wileyonlinelibrary.com]

### 1.4 Early Developments: Complementary Initiatives in North America and Europe

One of Europe's earliest organized initiatives was the founding of Kartagener Syndrom und Primäre Ciliäre Dyskinesie e.V. (PCD and KS e.V [40]) in Germany in 1997. From its inception, the association combined patient support and advocacy with active promotion of scientific research, close collaboration between patients and clinicians, and efforts to raise awareness of this under‐recognized disease. This partnership model became a defining feature of the pan‐European PCD community and anticipated the strong patient involvement that now characterizes international PCD research.

At the same time, major advances in molecular genetics were transforming the understanding of PCD, starting with the identification of *DNAI1* in 1999 [21] and *DNAH5* in 2002 [23], the latter remaining the most frequently mutated gene associated with PCD [41, 42, 43]. These landmark discoveries initiated a period of rapid gene identification that fundamentally changed the understanding of PCD pathogenesis and laid the foundation for subsequent advances in molecular diagnosis, genotype‐phenotype research, and precision medicine.

In North America, complementary developments focused on building coordinated clinical research infrastructure. Building on the early advocacy of the US PCD Foundation (PCDF [44]), founded in 2001 to address major unmet needs in diagnosis, treatment, research, and patient support, the Genetic Disorders of Mucociliary Clearance Consortium (GDMCC, Grant ID U54HL096458 [45]) was established in 2004 and NIH funded until 2026 as one of the original consortia within the Rare Diseases Clinical Research Network (RDCRN [46]). The consortium created a network of expert centers dedicated to longitudinal natural history studies, molecular genetics [47, 48, 49, 50, 51], biomarker validation, and detailed clinical phenotyping [52, 53, 54, 55, 56], providing one of the first coordinated clinical research infrastructures for PCD [39, 52, 57, 58].

Meanwhile, European collaboration expanded toward multinational programs on ciliary biology broadly. The EU FP7‐funded project EUCILIA (Pathophysiology of rare diseases due to ciliary dysfunction: nephronophthisis, Oral‐facial‐digital type 1 and Bardet‐Biedl syndromes, 2008–2011, Grant ID 201804 [59]) established ciliopathies as a unified disease group and developed experimental models informing motile ciliopathy research. Building on this, the EU‐FP7 funded project SYSCILIA (A systems biology approach to dissect cilia function and its disruption in human genetic disease, 2010–2015, Grant ID 241955 [60]) introduced systems biology to the field, mapping the ciliary proteome and interaction networks [61, 62] and contributing to the discovery of numerous ciliopathy‐associated genes, including several genes in which pathogenic variants are associated with PCD [63, 64, 65, 66, 67, 68, 69, 70, 71, 72, 73, 74, 75, 76, 77, 78, 79].

By the beginning of the 2010s, the two continents had established complementary strengths. North America had developed a coordinated clinical research infrastructure centered on patient advocacy [80], longitudinal cohorts, and natural history studies, while Europe had built internationally connected programs in molecular genetics and ciliary biology. In parallel, patient organizations on both continents increasingly linked with clinicians and researchers, supporting communication between the scientific and patient communities and facilitating collaborative research initiatives. Together, these developments laid the groundwork for the international PCD networks that emerged during the following decade.

### 1.5 Harmonizing Diagnosis and Building Collaborative Research

During the 2000s, advances in molecular genetics, ciliary biology, and clinical phenotyping highlighted the need for standardized diagnosis and coordinated research in PCD [81, 82, 83, 84].

Investigator‐driven collaborations between European and North American researchers became increasingly common, particularly in genetics and clinical phenotyping, including the characterization of *DNAI1* founder variants [47] and joint studies of laterality defects and congenital heart disease in PCD [54]. These early efforts demonstrated the value of combining expertise and cohorts across countries, fostered common diagnostic approaches, facilitated the exchange of biological samples and clinical data, and created lasting scientific partnerships that laid the scientific groundwork for the formal international networks that followed.

In parallel, the European Respiratory Society (ERS) convened a multidisciplinary Task Force from expert centers in 12 countries. Its 2009 consensus statement [85] provided the first coordinated pan‐European diagnostic and management framework, replacing local practice with harmonized recommendations [85, 86]. Importantly, the Task Force also defined a strategic research agenda, identifying standardized diagnostic pathways, early diagnosis, multicentre clinical trials, international patient registries, genotype‐phenotype studies, and evidence‐based treatment recommendations as priorities for future research [87].

Concurrently, GDMCC investigators established standardized clinical phenotyping and diagnostic protocols across participating centers and disseminated these through influential reviews synthesizing the rapidly expanding knowledge on PCD genetics, diagnosis, and clinical management [49, 52, 81, 82, 83, 88, 89]. Together, these activities provided a common clinical framework for the North American network while complementing the harmonization efforts taking place in Europe.

Though organizationally distinct, the European and North American initiatives increasingly converged through shared investigators, collaborative publications, and complementary scientific priorities. When the EU FP7‐funded BESTCILIA (Better Experimental Screening and Treatment for Primary Ciliary Dyskinesia, 2012–2016; Grant Agreement No. 305404 [90]) project was launched in 2012, it formalized an international scientific network that had already evolved through years of investigator‐driven collaboration across continents [23, 39, 47, 48, 54, 65, 66, 68, 88, 91, 92, 93, 94].

### 1.6 BESTCILIA: Establishing a Transatlantic PCD Research Infrastructure

Building on the priorities of the 2009 ERS Task Force and years of transatlantic collaboration, BESTCILIA marked a major step in the maturation of PCD research [90, 95]. It provided the first formal framework through which existing investigator networks were expanded and supported by shared infrastructure. The consortium brought together 11 expert centers from nine countries (seven European, two in the United States), to address longstanding evidence gaps and establish the infrastructure required for coordinated international research. It integrated complementary strengths developed independently on both sides of the Atlantic, North America's longitudinal natural history studies and standardized phenotyping, Europe's advances in molecular genetics and ciliary biology, within a common program of standardized data collection and collaborative studies.

A major achievement of BESTCILIA was the establishment of sustainable research infrastructure, prompted by a systematic review and meta‐analysis that revealed considerable heterogeneity in PCD clinical manifestations and underscored the need for larger prospective studies [96]: the prospective International PCD Registry and the retrospective international PCD (iPCD) Cohort [97, 98, 99], providing an unprecedented platform for epidemiological, genotype‐phenotype, and natural history studies while enabling clinical trial planning in substantially larger patient populations [97, 100, 101, 102, 103].

Standardized diagnostic protocols were implemented in Cyprus, Greece, and Poland, strengthening national diagnostic capacity, increasing the number of newly diagnosed PCD patients by 21.4%, and demonstrating that diagnostic expertise could be effectively transferred to previously under‐resourced countries [104]. These implementation efforts complemented BESTCILIA's broader contribution to strengthening the evidence base for PCD diagnosis and promoting international harmonization of diagnostic practice.

BESTCILIA generated the first robust evidence base for PCD diagnostics [105, 106, 107], contributing to the first evidence‐based ERS diagnostic guideline, which was developed by an international panel including North American experts within a second ERS Task Force (2014–2016) [108]. North American investigators subsequently developed the first American Thoracic Society (ATS) guideline, reflecting the evidence in the context of a different healthcare system and diagnostic pathway [109]. Although developed under different professional societies, the two guidelines showed substantial agreement while also highlighting areas of divergence [110]. Continued collaboration culminated in the joint ERS/ATS diagnostic guideline published in 2025 [111, 112].

BESTCILIA addressed another major unmet need by establishing the methodological infrastructure required for multinational clinical trials in PCD. The project developed disease‐specific quality‐of‐life instruments [113, 114, 115], evaluated outcome measures including multiple‐breath washout [116, 117], and established a validated framework for assessing therapeutic interventions. These advances directly enabled the first multinational randomized controlled trial in PCD, showing that long‐term azithromycin reduces respiratory exacerbations and establishing the feasibility of multicentre interventional trials in PCD [102, 103].

Beyond its scientific output, BESTCILIA established a durable collaborative framework linking clinicians, scientists, diagnostic specialists, epidemiologists, and patient organizations. Patient organizations, including the PCDF and PCD & KS e.V., participated as consortium partners and contributed to research planning, dissemination and patient engagement. This framework subsequently underpinned the BEAT‐PCD COST Action, the ERS Clinical Research Collaboration BEAT‐PCD, and other international initiatives that continue to advance clinical and translational PCD research.

### 1.7 From Collaborative Projects to Sustainable International Infrastructures

The completion of BESTCILIA marked the end of the first large‐scale transatlantic research project dedicated to PCD, but not the end of the scientific community it had strengthened. Instead, the long‐standing collaborations between European and North American investigators continued to evolve through complementary organizational structures that formalized research, clinical care, education, and patient engagement while maintaining the close transatlantic interactions that had characterized the field for many years.

### 1.8 BEAT‐PCD: Sustaining the International Research Community

The European Cooperation in Science and Technology (COST) Action BEAT‐PCD (Better Experimental Approaches to Treat Primary Ciliary Dyskinesia, BM1407, 2015–2019 [118]) was established to maintain and expand the collaborative framework developed through BESTCILIA. Rather than creating a new network, it brought together many of the same investigators together with additional clinicians, basic scientists, diagnostic specialists, epidemiologists, allied health professionals, statisticians, and patient representatives from Europe, North America, and beyond [119, 120, 121, 122].

Through thematic working groups it harmonized diagnostic approaches, clinical protocols, epidemiological methods, and research priorities, facilitating multicentre studies. A major contribution of the COST Action was the development of the community itself. Training schools, workshops, short‐term scientific missions, and support for early‐career investigators helped disseminate expertise throughout the expanding PCD network and fostered a new generation of researchers and clinicians. Scientific exchange was further strengthened through dedicated annual international meetings [119, 120, 121, 122, 123].

To ensure continuity beyond the duration of the COST Action, the European Respiratory Society (ERS) Clinical Research Collaboration (CRC) BEAT‐PCD was launched in 2020 [124]. Building directly on the scientific networks established through BESTCILIA and the COST Action, the CRC now provides a long‐term framework for multinational clinical and translational research under the auspices of the ERS.

### 1.9 ERN‐LUNG: Institutionalizing Expert Clinical Care

While BEAT‐PCD focused on collaborative research, the establishment of the European Reference Networks (ERNs) by the European Commission in 2017 created a complementary clinical infrastructure for rare diseases [125, 126]. Following a rigorous independent assessment of clinical expertise, multidisciplinary organization, diagnostic capability, research activity, and quality management, healthcare providers were designated by the Board of Member States as members of the ERN. This process created a network of accredited expert centers capable of delivering highly specialized care and cross‐border care, developing clinical practice guidelines, supporting education and training, and contributing to collaborative research [125, 126, 127, 128].

Within the ERN for rare respiratory diseases (ERN‐LUNG) [129, 130, 131], the PCD Core Network built directly on the collaborations established through BESTCILIA and BEAT‐PCD, aiming on harmonizing diagnostic pathways and standards of care, strengthening registries, and integrating research with clinical practice. A major achievement has been the establishment of the Primary Ciliary Dyskinesia Clinical Trial Network (PCD‐CTN) [132, 133], extending this infrastructure into a dedicated multinational platform for therapeutic studies through standardized site qualification, harmonized trial procedures, and close collaboration with patient organizations, academic investigators, and industry.

### 1.10 Development of the North American Network

Throughout this period, the North American PCD community continued to evolve through complementary organizational structures. Building on the NIH‐funded GDMCC [45], the PCDF assumed a central coordinating role coordinating clinical care, research, education, and patient advocacy. Under its leadership, the PCD Foundation Clinical and Research Centers Network (PCDF CRCN) was established in 2012 to provide patients with access to specialized PCD diagnosis and care. After accreditation procedures, training and a vetting process had been set up, the first site was accredited in 2015. Since then, the network has grown into a group of centers committed to standardized diagnosis, multidisciplinary care, longitudinal follow‐up, research and education.

Building on this clinical and research infrastructure, the PCD Foundation Network for Clinical Trials (PCDF NCT) [134] was subsequently established to support investigator‐initiated and industry‐sponsored therapeutic studies. Together with the European PCD Clinical Trial Network [132, 133], these complementary initiatives have created robust infrastructures for multinational interventional research while continuing the close scientific interactions that have long characterized the transatlantic PCD community [90, 132].

Though developed within different organizational and funding structures, European Commission and ERS initiatives versus NIH‐ and PCD Foundation‐supported programs, the two networks have converged toward similar goals: integrating patient organizations, building expert clinical center networks, developing standardized diagnostic pathways and registries, and creating infrastructure for multinational trials, together providing the basis for an increasingly coordinated global approach to PCD research and therapeutic development.

### 1.11 Rare Disease Registries: From Natural History to Trial Readiness

The increasing international collaboration in PCD research also highlighted the need for standardized longitudinal clinical data. Despite regular follow‐up of patients in specialized centers, most information remained confined to local medical records, limiting opportunities to study the natural history of this rare and genetically heterogeneous disease or to evaluate diagnostic and therapeutic strategies across larger patient populations. During the early 2000s, the North American GDMCC established prospective natural history studies that became a cornerstone of clinical research in PCD. Building on the collaborative infrastructure created through BESTCILIA, Europe subsequently established the retrospective international PCD (iPCD) Cohort [98] and the prospective International PCD Registry [97], providing the first multinational platforms for harmonized data collection across expert centers. These pioneering initiatives demonstrated that standardized multinational registries were both feasible and essential for rare disease research. By harmonizing longitudinal clinical and genetic data across expert centers on international level, they substantially advanced the study of disease epidemiology, natural history, genotype‐phenotype relationships, and long‐term outcomes [42, 135]. Equally importantly, they provided the evidence base for clinical guidelines, biomarker research, and the development of outcome measures required for future therapeutic trials [42, 100, 136, 137, 138, 139, 140].

Over the past decade, numerous national and international registries and longitudinal cohort studies have been established in both Europe and North America [97, 98, 99, 141, 142, 143, 144], reflecting the continued maturation of the international PCD community. While differing in governance, methodology, and geographical scope, these complementary initiatives share the common objective of generating high‐quality real‐world evidence and supporting collaborative research across national borders. Today, PCD registries have evolved far beyond observational databases. They have become essential components of the international PCD research infrastructure that facilitate patient recruitment, support multinational investigator‐initiated and industry‐sponsored clinical trials, and increasingly provide the foundation for genotype‐specific and individualized therapeutic approaches. In addition to center‐based enrollment, patients can also register themselves directly in patient‐driven registries such as the PCDF Connect Registry of the US PCD Foundation [142] and the ERN‐LUNG BREATHeRegistry [144] for rare respiratory diseases. These registries allow individuals with a confirmed, highly likely, or clinical diagnosis of PCD, or, in the case of the BREATHe Registry also any other rare respiratory disease, to enroll regardless of where they receive clinical care, connecting them with expert centers, clinical trials, researchers, and patient organizations worldwide.

### 1.12 Disease‐Specific Versus Syndrome‐Based Registries

Established in 2012 as one of the first European Respiratory Society Clinical Research Collaborations (ERS CRCs), the European Multicentre Bronchiectasis Audit and Research Collaboration (EMBARC [145]) has developed into the largest international research network dedicated to bronchiectasis. Through its multinational registry [146] and associated translational research programs, EMBARC has generated valuable real‐world data on disease epidemiology, microbiology, clinical outcomes, and treatment response [147, 148, 149, 150, 151, 152].

EMBARC [146] and EMBARC aligned registries such as the German Bronchiectasis Registry PROGNOSIS [153], the Indian Bronchiectasis Registry (EMBARC‐India) [154], Chinese Bronchiectasis Registry (BE‐China [155]), or the Australian Bronchiectasis Registry (ABR) [156] follow a syndrome‐based approach, enrolling patients with bronchiectasis of any etiology except cystic fibrosis and lung transplant. While this allows shared clinical characteristics and treatment responses to be studied across large and heterogeneous populations [152, 157, 158, 159], it also means that the underlying cause of bronchiectasis often remains unknown despite advances in genetic analyses [156, 160, 161]. PCD registries take a fundamentally different, disease‐specific approach: patients are enrolled only after a confirmed diagnosis of PCD, established according to standardized diagnostic criteria [97, 98, 99, 143]. This yields deeply phenotyped, biologically defined cohorts in which molecular defects, ciliary dysfunction, and clinical outcomes can be directly and reliably linked to the underlying molecular and genetic defect [42, 54, 136, 138, 162, 163, 164, 165, 166, 167, 168, 169, 170, 171, 172, 173, 174], a level of etiological certainty that syndrome‐based registries such as EMBARC can currently not offer.

This distinction is not merely academic but has direct translational relevance. Because the underlying disease mechanism in PCD is known, PCD registries make it possible to determine whether therapeutic responses differ according to cause, a question EMBARC's heterogeneous, largely idiopathic or mixed‐etiology cohort cannot answer with the same precision. The two approaches are therefore complementary: EMBARC is ideally suited to studying bronchiectasis as a shared clinical syndrome, whereas disease‐specific PCD registries remain indispensable whenever research questions, particularly in translational studies and clinical trials, depend on accurately diagnosed, mechanistically defined patient populations.

### 1.13 Scientific Meetings and Community Building

Patient organizations have been integral to the international PCD research landscape from the outset, contributing as active partners to its scientific, clinical, and advocacy efforts [80, 108, 111, 132, 175, 176, 177, 178, 179, 180, 181, 182, 183, 184, 185, 186]. In the United States, the PCDF established the annual “PCD on the Move” meetings in 2014, a major forum for disseminating advances in basic science, clinical research, and patient care, and a training environment for clinicians, researchers, and patient representatives. In parallel, the annual COST BEAT‐PCD meetings in Europe, established in 2015, were replaced from 2022 onward by PCD‐specific meetings held immediately prior to the annual European Respiratory Society (ERS) Congress (“pre‐ERS PCD meetings”), organized jointly by the ERS CRC BEAT‐PCD and the ERN‐LUNG PCD Core, which, similar to “PCD on the Move,” became one of the key venues for coordinating international PCD research and involved patient representatives alongside clinicians and scientists. As the pace of basic, clinical, and translational research in PCD accelerated, a dedicated, multi‐day international congress format has been established by the ERN‐LUNG PCD Core. Meetings in Cyprus and Naples brought together clinicians, scientists, allied health professionals, and patient organizations to discuss diagnostics, clinical care, and translational research, aligning European activities with the wider global effort and strengthening international partnerships.

Just as the scientific and clinical networks converged over time, patient organizations themselves developed a parallel history of connection and collaboration. With the Kartagener Syndrom und Primäre Ciliäre Dyskinesie e.V. founded in 1997 [40], followed a few years later by the PCD Foundation in the United States [44], a growing network of national patient associations emerged across Europe, Australia, and beyond over the following two decades (Table 1), together with umbrella bodies such as the European Lung Foundation (ELF) [187]. These organizations not only provided patients and families with peer support, information, and a collective voice in research and policy, but increasingly connected with one another and with the clinical and research networks described above, fostering joint projects and shared advocacy. Since 2021, annual online patient congresses organized by BEAT‐PCD have explicitly linked these national communities, mirroring at the patient level the same drive toward coordinated structures seen among the scientific networks.

**TABLE 1:** International PCD clinical care networks, research collaborations, registries and patient organizations.

	Country/Region	Website
Clinical Care and Research Networks		
ERN‐LUNG	Europe	https://ern-lung.eu/
ERN‐LUNG PCD Core	Europe	https://ern-lung.eu/our-expertise/disease-groups/pcd-about/
PCD Foundation Clinical and Research Centers Network (PCDF CRCN)	USA	https://www.pcdfoundation.org/what-are-clinical-centers/
Clinical Research Collaboration		
ERS CRC BEAT‐PCD	Europe	https://beat-pcd.squarespace.com/
Clinical Trial Networks		
ERN‐LUNG Clinical Trial Network for PCD (PCD‐CTN)	Europe	https://ern-lung.eu/our-expertise/disease-groups/pcd-about/pcd-clinical-trial-network/
PCDF Network for Clinical Trials (PCDF NCT)	USA	https://pcdfoundation.org/research-2/the-primary-ciliary-dyskinesia-foundation-network-for-clinical-trials/
International PCD Registries		
International PCD Registry	Europe	https://www.pcdregistry.eu/
Follow‐up	Europe	Goutaki et al. ERJ Open Research 2020 6: 00237‐2019
PCD Foundation Registry	USA	https://www.pcdfoundation.org/registry/
Patient‐driven engagement & data platforms		
ERN‐LUNG BREATHeREGISTRY	Europe	https://ern-lung.eu/patient-registry/breatheregistry/
PCDF Connect	USA	https://www.pcdfoundation.org/pcdfconnect/
Patient associations		
ADCP Association	France	https://www.adcp.asso.fr/
Asociación Nacional de Pacientes con Discinesia Ciliar Primaria (DCP España)	Spain	https://dcpes.org/
Associazione italiana discinesia ciliare primaria – sindrome di Kartagener Aps	Italy	https://www.pcdkartagener.it/
Breathing Together	Cyprus	https://www.pcdcyprus.com/
Dyskinesia A.S.B.L.	Belgium	https://www.facebook.com/asbl.dyskinesia
Hengitysliitto ry	Finland	https://www.hengitysliitto.fi/
Kartagener Syndrom und Primäre Ciliäre Dyskinesie e.V.	Germany, Austria, Switzerland	https://www.pcd-ks.info/
Lung Foundation Australia	Australia	https://lungfoundation.com.au/
Norsk forening for cystisk fibrose (PCD)	Norway	https://www.cfnorge.no/
PCD Australia	Australia	https://www.pcdaustralia.org.au/
PCD Belangengroep	Netherlands	https://www.p-c-d.org/
PCD Foundation (PCDF)	USA	https://www.pcdfoundation.org
PCD Support UK	United Kingdom	https://pcdsupport.org.uk/
PCD Sverige	Sweden	https://www.pcdsverige.se/
Polskie Towarzystwo Dyskinezy Rzęsek	Poland	http://ptdr.pl/
Primary Ciliary Dyskinesia Association Georgia	Georgia	https://www.facebook.com/PCD.Diskinezia.Kartagener
Primer Siliyer Diskinezi Derneği (SiLYADER)	Turkey	https://silyader.org.tr/
Riksförbundet Cystisk Fibros (RfCF)	Sweden	https://www.rfcf.se/
RunningOnAir	USA	https://runningonair.net/
Řasinka z. s.	Czech Republic	https://www.spolekrasinka.cz/
Първична цилиарна дискинезия/Синдром на Картагенер	Bulgaria	https://www.facebook.com/profile.php?id=100067189670503&sk=about
Patient‐driven alliances of patient organizations		
European Lung Foundation (ELF)	Europe	https://europeanlung.org/
EURORDIS	Europe	https://www.eurordis.org/

A major milestone reflecting this convergence on both fronts is the joint initiative of the PCDF, ERN‐LUNG PCD Core, and ERS CRC BEAT‐PCD to establish the Global PCD Congress series (Figure 1), the first of which was held in Montreal, Canada, in 2026 with the second scheduled for Lisbon, Portugal, in October 2027. It was the first meeting jointly organized by the three principal organizations that have shaped the field's international development over the past two decades. It was attended by 327 participants from 24 countries across Europe, North and South America, Asia, and Australia, among them 97 people with PCD and their family members, as well as a markedly increased number of industry partners and sponsors. A subsequent summarizing webinar reached a further 121 participants from 23 countries.

The program spanned the full translational spectrum: new diagnostic and management guidelines, presented alongside patient accounts of daily life with the disease and of expectations regarding the growing number of clinical trials; advances in diagnostics, including long‐read sequencing and artificial intelligence applied to genetic, imaging, and ciliary motility data, as well as new animal models for functional and preclinical studies; the activities of the collaborating networks and their registries together with extrapulmonary manifestations such as situs anomalies, hydrocephalus, infertility, and ENT disease; and finally outcome measures, among them radioaerosol assessment of mucociliary clearance, together with results from completed and ongoing clinical trials in PCD, spanning a mutation‐specific inhaled mRNA therapy and anti‐inflammatory compounds. Most known PCD patient organizations likewise took part, discussing the future of patient community building and joint activities, reflecting the continued evolution of transatlantic collaboration into coordinated organizational structures on both the scientific and patient sides.

### 1.14 Training the Next Generation

A less visible but arguably more durable outcome of these collaborations has been the training of a new generation of PCD researchers, clinicians, and other healthcare professionals, pursued from the outset as one of the networks' stated objectives. BESTCILIA embedded doctoral students and clinical fellows in multicentre work [90], and the COST Action BEAT‐PCD [118, 119, 120, 121, 122] made capacity building an explicit objective, holding successive PCD training schools co‐located with its annual conferences and funding short‐term scientific missions through which trainees acquired specialized diagnostic and laboratory techniques in centers that had established them. These activities have been continued and institutionalized: early career researchers hold designated seats in the governance of the ERS Clinical Research Collaboration BEAT‐PCD [118, 188], the ERN‐LUNG Academy [189] offers structured webinars, e‐cases and observational stays at expert centers together with joint ERS/ERN‐LUNG fellowships, and the PCD Foundation [44, 190] combined its annual scientific conferences, which serves explicitly as a training meeting for new investigators, with dedicated early career investigator funding. Many of those trained through these routes now lead PCD services and research programs in their own countries, several of which previously had no local expertise. Sustaining this pipeline beyond individual funding periods will be critical if PCD research and care are to continue developing over the coming decades.

### 1.15 From Networks to Recognition

These collaborative structures have had an effect beyond the research questions they were designed to address. The disciplines engaged with PCD have broadened well beyond pediatric pulmonology to include adult respiratory medicine, otorhinolaryngology, cardiology, reproductive medicine, human genetics, radiology, physiotherapy and specialist nursing, a shift visible in the multidisciplinary authorship of recent guidelines and standards documents [54, 85, 111, 119, 186, 191, 192, 193, 194]. Registries have expanded from single‐center cohorts to harmonized international data collection, a process that has itself prompted participating centers to identify and review their patient populations [42, 87, 97, 98]. The meetings and training activities described above have provided a recurring international forum and, through training schools, short‐term scientific missions and fellowships, have been instrumental in establishing PCD expertise in centers that previously had none. Together, these developments have raised the visibility of the disease among clinicians, funders and health authorities, although awareness remains concentrated in participating countries and diagnostic delay persists [87, 176, 195, 196].

## 2 Conclusion: Advances and Open Challenges

International collaboration has fundamentally transformed the field of PCD and related ciliopathies. The convergence of registries, clinical trial networks, and reference networks is creating a highly interconnected research ecosystem that enables efficient data sharing and accelerates translational research, while international collaborations have simultaneously driven significant progress in standardizing diagnostic criteria and clinical outcome measures, improving comparability across studies. Building on this foundation, advances in genetic characterization and large‐scale data analysis are paving the way for personalized approaches to diagnosis and treatment. Importantly, genetic analysis needs to be more broadly implemented to identify the underlying genetic cause in PCD and bronchiectasis individuals, enabling accurate diagnosis and paving the way for mechanism‐based, precision medicine approaches. Patient organizations, involved from the outset, are increasingly connecting and joining forces across countries and continents, forming international alliances that amplify the patient voice, harmonize advocacy efforts, and ensure that research priorities, study design, and dissemination of findings are shaped by the lived experience of those affected worldwide. Coordinated efforts spanning registries, clinical networks, and trial platforms are thus moving the field toward evidence‐based and precision medicine approaches. Closer connections with the heterotaxy community would also be valuable, given the partial overlap in ciliary mechanisms between PCD and laterality defects.

Despite this substantial progress, challenges remain, including limited representation of low‐ and middle‐income countries and unequal access to diagnostic tools [177, 197, 198, 199, 200, 201, 202, 203]. In many countries, a definitive diagnosis currently depends on bilateral collaborations in which patient samples, for example blood for genetic testing and respiratory epithelial cells for molecular and functional analyses, are shipped to specialized centers abroad, an approach that relies largely on the commitment and resources of individual research groups. For larger studies, the International PCD Registry records, for consenting participants, whether biological material is available and of which type, and whether it is linked to a local biobank, thereby providing an index of existing, physically decentralized collections. What is lacking is the infrastructure beneath this: funding for transport, processing, analysis, long‐term storage and curation, personnel for sample management, and a guarantee of sustainability beyond individual project funding. Establishing such infrastructure, whether centrally or as a federated structure analogous to the standardized and networked approaches already adopted for clinical and genetic data, together with harmonized standard operating procedures for sample collection, storage and shipment and workable legal and ethical frameworks for cross‐border sample and data sharing, therefore represents an important unmet need, ideally combined with capacity building to strengthen local diagnostic capabilities.

Future efforts should therefore focus on expanding global inclusivity, building sustainable bioresource infrastructures, strengthening trial capacity, and deepening interdisciplinary research across PCD and other motile ciliopathies and mucociliary clearance disorders, while further building ties to bronchiectasis research as a shared but etiologically diverse clinical endpoint.

## Author Contributions

**Petra Pennekamp:** conceptualization, writing – review and editing, writing – original draft. **Michele Manion:** conceptualization, writing – review and editing. **Heymut Omran:** conceptualization, writing – review and editing.

## Conflicts of Interest

The authors declare no conflicts of interest.

## Supporting information


Supporting File

## Data Availability

Data sharing not applicable to this article as no datasets were generated or analyzed during the current study.
