# Supplementary material for: International Collaboration in Primary Ciliary Dyskinesia
Source: Pediatr Pulmonol. 2026 Oct 28;61(11):e71888. doi: 10.1002/ppul.71888 (PMC13717290; doi:10.1002/ppul.71888)
Supplement: Supplementary file 1 [file PPUL-61-0-s001.pdf]

# Supplementary file

### Search strategy

This is a narrative review informed primarily by the authors' first-hand knowledge of and long-standing involvement in the field of primary ciliary dyskinesia (PCD) research and international collaboration.

Given the historical and interdisciplinary scope of this review, no systematic or structured search protocol (e.g., PRISMA) was applied. Instead, sources were selected based on relevance to the narrative and the authors' expert knowledge of the field.

This experiential knowledge was complemented by targeted literature searches. Peer-reviewed publications were identified and verified via PubMed, using search terms including, among others: primary ciliary dyskinesia, PCD, collaboration in rare diseases, Beat-PCD, ERN-LUNG, PCD Foundation, bronchiectasis, Registr*, genotype-phenotype correlation. Additional publications were included by screening the reference lists from included papers or reviews.

Additional information on funding programs, project reports, and institutional activities was obtained through general web searches and relevant funding agency websites; specific sources are provided as links in the reference list.
